# Psychosocial stress and immunosuppression in cancer: what can we learn from new research?

**DOI:** 10.1192/bja.2021.9

**Published:** 2021-04-23

**Authors:** Anurag K. Singh, Udit Chatterjee, Cameron R. MacDonald, Elizabeth A. Repasky, Uriel Halbreich

**Affiliations:** Department of Radiation Medicine, Roswell Park Comprehensive Cancer Center, Buffalo, NY, USA.; Department of Radiation Medicine, Roswell Park Comprehensive Cancer Center, Buffalo, NY, USA.; Department of Immunology, Roswell Park Comprehensive Cancer Center, Jacobs School of Medicine and Biomedical Sciences, Buffalo, NY, USA.; Roswell Park Comprehensive Cancer Center, Buffalo, NY, USA; Department of Psychiatry, Jacobs School of Medicine and Biomedical Sciences, University at Buffalo, NY, USA.

**Keywords:** Anxiety disorders, depressive disorders, epidemiology, neuroendocrinology, neuroimmunology

## Abstract

It is generally believed that the physiological consequences of stress could contribute to poor outcomes for patients being treated for cancer. However, despite preclinical and clinical evidence suggesting that stress promotes increased cancer-related mortality, a comprehensive understanding of the mechanisms involved in mediating these effects does not yet exist. We reviewed 47 clinical studies published between 2007 and 2020 to determine whether psychosocial stress affects clinical outcomes in cancer: 6.4% of studies showed a protective effect; 44.6% showed a harmful effect; 48.9% showed no association. These data suggest that psychosocial stress could affect cancer incidence and/or mortality, but the association is unclear. To shed light on this potentially important relationship, objective biomarkers of stress are needed to more accurately evaluate levels of stress and its downstream effects. As a potential candidate, the neuroendocrine signalling pathways initiated by stress are known to affect anti-tumour immune cells, and here we summarise how this may promote an immunosuppressive, pro-tumour microenvironment. Further research must be done to understand the relationships between stress and immunity to more accurately measure how stress affects cancer progression and outcome.

The complex interplay of known genetic, environmental, lifestyle and endocrine factors leading to carcinogenesis, cancer progression and the response to therapies is challenging to model (Colditz 2012). Socioeconomic factors have also been implicated in cancer incidence and outcomes (Aghdam 2020; Unger 2020) and factors such as psychological and physical stress are increasingly a source of concern for patients and their caregivers. But whether and how stress and/or biobehavioural and psychological responses to stressful circumstances play a role in cancer incidence and progression remain topics for which much more research is needed. Galen, the ancient Roman physician, posited that cancer was a constitutional disease more likely to afflict the melancholic (Hajdu 2004), and modern researchers continue to study the contribution of psychosocial factors to cancer incidence and survival. Chida et al performed a meta-analysis of the literature in 2007, and they found that stress-related psychosocial factors were correlated with a higher cancer incidence and poorer survival/higher cancer mortality (Chida 2008a). Despite this, last updated in 2012, the National Cancer Institute felt that the evidence linking stress and cancer incidence is weak, and we believe this connection should be more comprehensively studied (National Cancer Institute 2012).

Since these reports, the impact of stress on cancer progression, response to therapies and overall outcome has gained considerable new interest. Emerging preclinical and clinical studies have elucidated mechanisms of cross-talk between the nervous system and the body -n tumorigenesis (Magnon 2013) and outcomes (Mauffrey 2019). In particular, recent work has revealed many of the mechanisms underlying the ability of neuroendocrine stress pathways, such as the hypothalamic–pituitary–adrenal (HPA) axis, the sympathetic nervous system (SNS) and β-adrenergic signalling, to mediate the immune system’s capacity to detect and destroy malignant cells (Chen 2020). This developing understanding of the nervous system–stress signalling–immune system axis as a major contributor to treatment outcomes may open new avenues for studying the role of psychosocial stress on cancer incidence and survival/mortality.

To help with our own understanding of where the field of research on psychosocial stress-related factors and cancer stands, we performed a systematic review of the literature since 2007. We found that there are as many studies that show a relationship between stress and cancer as those that show no relationship. However, in comparison, very few studies show a beneficial effect of stress on cancer outcomes. We hope that the accompanying table (supplementary Table 1 and supplementary file 1, available at https://doi.org/10.1192/bja.2021.9) of information regarding that literature can be of use, or a starting point for other investigations. Additionally, we identified new findings describing the roles many immune cells play in cancer progression and response to therapy, and summarised how HPA- and SNS-mediated stress significantly influence this anti-tumour immune response. Importantly, there is now strong evidence that these chronic stress pathways lead to an impairment of anti-tumour immune cells and an enhancement of pro-tumour immunosuppressive cells.

We believe that it will be extremely relevant to consider the impact of stress on these anti- and pro-tumour immune cells, which culminate in a suppressed immune response to malignant cells. In the context of anti-tumour immunity, further breakthroughs in this field may yield important new biomarkers and other tools for the more objective assessment of levels of stress in patients. Additional discoveries could provide a rationale for novel methods of augmenting anti-tumour immunity and improving cancer therapies that depend on generating or improving the immune response to cancer.

## Analysis of the literature related to the role of stress in cancer

**FIG 1:**
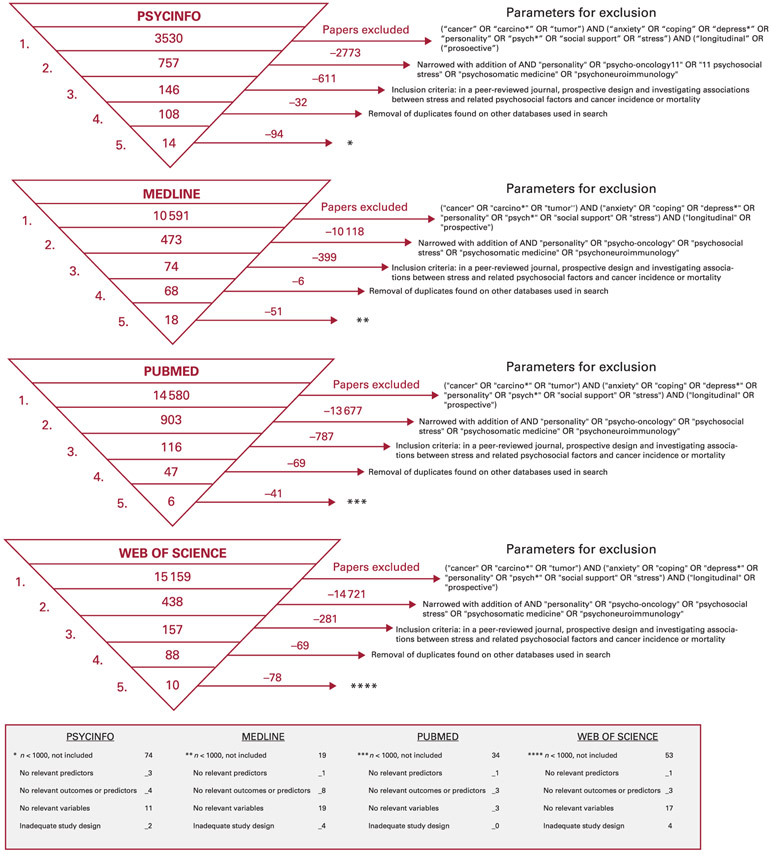
Parameters for the literature search.

We searched the literature using the same search parameters as Chida et al (Chida 2008a) and we used the MEDLINE, PsycInfo, Web of Science and PubMed databases for articles published between 2 October 2007 and 1 April 2020. Specifically, we searched for: (‘cancer’ OR ‘carcino*’ OR ‘tumor’) AND (‘anxiety’ OR ‘coping’ OR ‘depress*’ OR ‘personality’ OR ‘psych*’ OR ‘social support’ OR ‘stress’) AND (‘longitudinal’ OR ‘prospective’). Although the individual patient data were not available for our analysis, we used the reported hazard or odds ratios. Criteria for inclusion were publication of papers in the English language in a peer-reviewed journal, with a prospective study design and investigating associations between stress and related psychosocial factors and cancer incidence or mortality. Studies with fewer than 1000 participants were excluded from further analysis. Other criteria related to the analysis are described in Fig. 1.

In total, 47 studies were selected for further review (summarised in supplementary Table 1). We categorised studies only by the hazard or odds ratios presented. Thus, in some cases (such as White et al (2007), Archer et al (2015) and Nakaya et al (2010)) the findings are listed as significant although the authors themselves discounted their findings. Similarly, in a few cases the authors used alternative analyses that showed a significant result that was not supported by the hazard ratios and therefore are not listed as significant in the table.

Only 3 studies (6.4%) showed a protective effect of psychosocial stress on either cancer incidence or mortality. A harmful effect of psychosocial stress on cancer incidence and/or mortality/survival was found in 21 of the 47 studies (44.6%.) The remaining 23 studies (48.9%) showed no association between psychosocial stress and cancer. Taken together, these data certainly suggest that psychosocial stress may have an impact on cancer incidence and/or mortality. However, significant associations were found in approximately half of the studies. We suggest that this rate of significant association may be improved by better linking the perceived stress with measurable physiological alterations produced by the systemic biological effects of that stress (for example on the immune system.) Interestingly, positive psychological traits were associated with reduced HPA reactivity (Chida 2008b). Consistent with this finding, Cole et al, in rhesus macaques, found that chronic social stress alters endogenous glucocorticoids, which impairs normal physiological regulation of leukocyte function by the HPA axis (Cole 2009). Adam et al performed a meta-analysis of 80 studies and found that flatter diurnal cortisol slopes lead to worse health outcomes and showed strong effects on immune and inflammatory outcomes (Adam 2017).

## New research directions likely to help clarify the full impact of stress on cancer

When considering possible mechanisms that could be driving the clinical observation that chronic stress likely leads to poorer overall outcomes for patients being treated for various malignancies, it is important to consider the implications of chronic stress on the anti-tumour immune system, which will be discussed next Through various neuroendocrine signalling pathways, primarily the SNS and the HPA axis, mediators of stress responses culminate in a generally impaired immune system, characterised by specific changes in activity of various cell types, including, but not limited to: natural killer cells, dendritic cells, CD8^+^ cytotoxic T cells, helper T (Th) cells, regulatory T cells (Tregs), myeloid-derived suppressor cells (MDSCs) and macrophages.

As the classic mediator of the fight or flight response, the SNS plays a critical role in pushing physiology outside of normal homeostatic parameters to allow for the avoidance of acute threats. After the release of noradrenaline and adrenaline into the bloodstream, these mediators then bind adrenergic receptors present throughout the body and influence processes such as heart rate and blood pressure, causing them to increase, while gastrointestinal function and the immune system are temporarily deprioritised and reduced. In the case of many patients being treated for cancer, the stress that occurs after a diagnosis typically becomes chronic and leads to long-term activation of this SNS stress response. In addition to potentially having negative consequences on aspects of health related to cardiovascular disease (Spruill 2010), the immunosuppressive potential of this SNS-driven adrenergic stress could potentially play an important role in cancer outcome.

**FIG 2:**
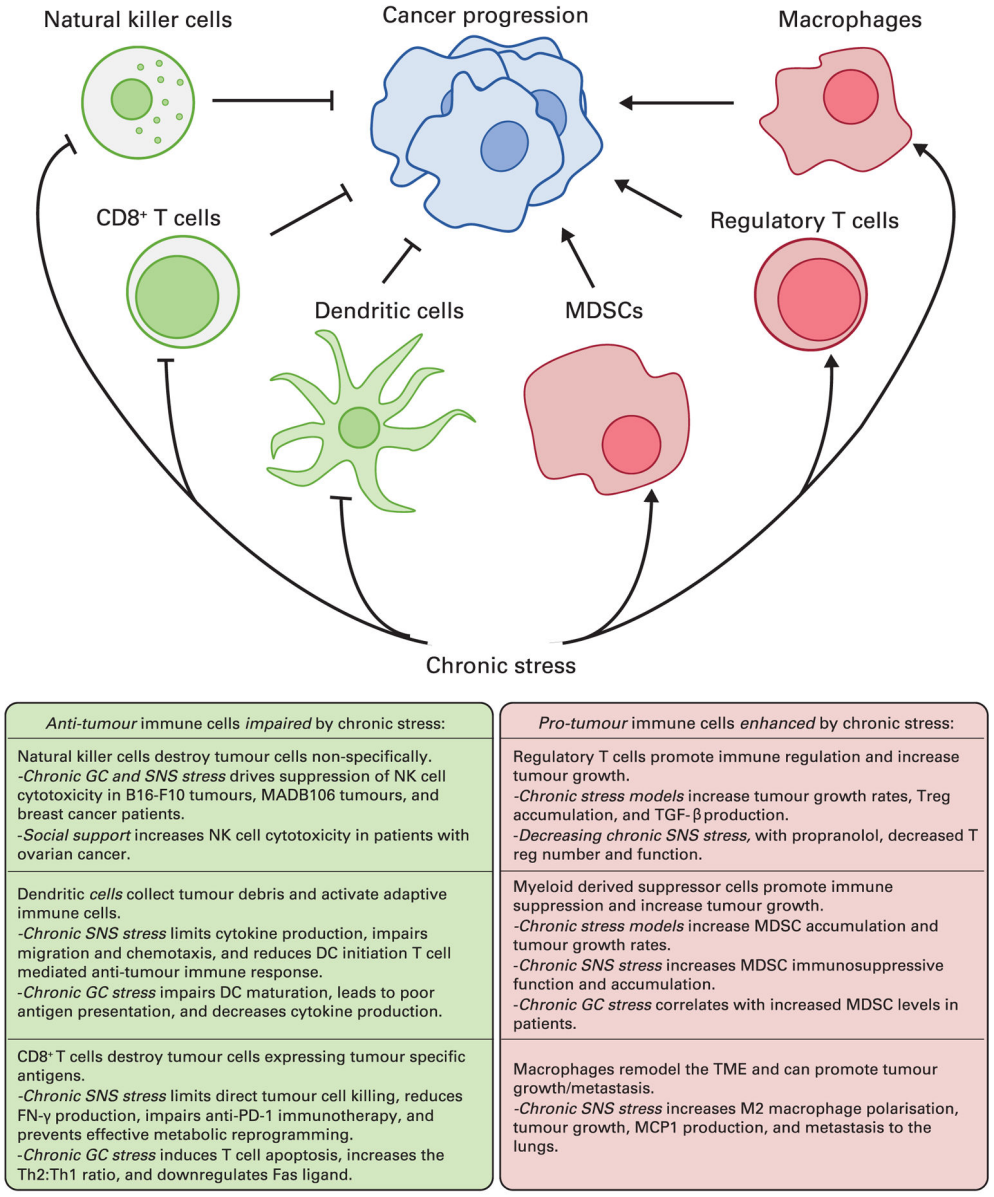
Summary of key immune cells and the general pro-tumour effects of chronic stress. MDSC, myeloid-derived suppressor cell; NK, natural killer; TGF-β, transforming growth factor beta; Treg, regulatory T cell; SNS, sympathetic nervous system; DC, dendritic cell; GC, glucocorticoid; TME, tumour microenvironment; IFN-γ, interferon-gamma; Th, helper T cell.

In addition to the SNS response, the production of glucocorticoids by the HPA axis can also have diverse effects on the body and can be driven by chronic psychological stress. These effects include metabolic, vascular, gastrointestinal and ocular changes, but one major outcome of chronic glucocorticoid stress signalling is immunosuppression. Clinically, the immunosuppressive properties of glucocorticoids make them useful therapeutics treating various disorders of immune overactivation. Similarly, much of the work done studying the impact of glucocorticoids on cancer has focused on using them to treat lymphoproliferative disorders such as lymphomas and leukaemias (McKay 2003), rather than studying their immunosuppressive consequences on anti-tumour immunity. More recent research has begun to tease apart the variable effects that glucocorticoid signalling can have in the context of different cancers, bringing light to a number of complex interactions promoting various pro- and/or anti-tumour processes (Lin 2016). The following is a brief summary of recent literature regarding the effects of stress hormones and stress signalling on cells associated with the immune response to cancer. This information is also summarised in Fig. 2.

### Natural killer cells

Natural killer cells are the primary anti-tumour cell type of the innate immune system. To differentiate between healthy and malignant cells, natural killer cells use various cell surface receptors to interrogate host cells and assess them for signs of viral infection or dysregulation common to cancer cells. If aberrant production of protons is occurring in a cell, abnormal cell-surface proton expression profiles are detected by natural killer cells, and this initiates a series of processes that culminate in the destruction of the identified tumour cell (Moretta 2002). Additionally, natural killer cells have the ability to target and destroy tumour cells coated with antibodies, in the event that a humoral immune response to a tumour antigen has occurred (Malmberg 2017).

For a natural killer cell to kill a cancer cell, two mechanisms are primarily employed. First, the detection of a dysregulated or antibody-coated cell triggers the release of several effector molecules, including perforin and granzymes (Voskoboinik 2015). Perforin creates small pores in the cellular membrane of the targeted tumour cell, and granzymes can then pass through the holes made by perforins (Voskoboinik 2015). From there, granzymes initiate apoptotic signalling pathways through caspase cleavage, which leads to the death of the tumour cell (Voskoboinik 2015). The second major mechanism that natural killer cells use to induce cell death is through tumour necrosis factor (TNF)-related apoptosis-inducing ligand (TRAIL) signalling (Prager 2019). TRAIL is a membrane-bound protein on the surface of natural killer cells that binds the death receptors DR4 and DR5 (Prager 2019). If present on a tumour cell, death-receptor signalling then leads to apoptosis through caspase cleavage in a mechanism somewhat similar to granzyme-induced apoptosis (Prager 2019).

Although acute sympathetic activation, such as that observed during exercise, may be beneficial for improving natural killer cell mobilisation and function (Schedlowski 1993; Idorn 2016; Pedersen 2016), early studies assessing the impact of repeated activation of β_2_-adrenergic receptors (β_2_-AR) on natural killer cells found it to be immunosuppressive and to lead to progression of a natural killer cell-sensitive tumour model (Shakhar 1998). As more sophisticated methods of investigation have developed, an increasing body of evidence suggests that exposure to physiologically relevant amounts of chronic stress signalling leads to suppression of natural killer cell cytotoxicity. These studies found that causing stress in mice or rats by various methods resulted in an increase in both sympathetic stress and glucocorticoid stress, which resulted in a decrease in killing of B16-F10 melanoma cells (De Lorenzo 2015) and MADB106 tumour cell clearance (Rosenne 2014) respectively. Interestingly, on further interrogation, it was discovered that glucocorticoid signalling drove an increase in β_2_-AR expression, thus increasing the sensitivity of natural killer cells to sympathetic stress (Rosenne 2014). Importantly, pharmacological inhibition of adrenergic signalling reversed the effect of not only sympathetic suppression, but also glucocorticoid suppression, suggesting that adrenergic signalling contributed most significantly to this phenomenon (Rosenne 2014).

Work from Thornton et al (2007) evaluated natural killer cell cytotoxicity over time in women diagnosed with breast cancer, and they found that women who reported a more rapid reduction in stress in the weeks after diagnosis showed a greater improvement in natural killer cell cytotoxicity compared with women who reported a slow decline in stress levels (Thornton 2007). When cytotoxic activity was assessed in natural killer cells from the tumour microenvironment and blood of women being treated for ovarian cancer, it was also found that high social support correlated with increased cytotoxicity, and self-reported distress was associated with decreased cytotoxicity (Lutgendorf 2005).

### Dendritic cells

Dendritic cells are another innate immune cell type that play an essential role in the generation of an anti-tumour immune response and are negatively affected by stress. As a diverse group of professional antigen-presenting cells, dendritic cells are responsible for sampling antigens present in the tumour microenvironment and initiating a robust adaptive immune response (Wculek 2020). During antigen uptake, dendritic cells assess the cytokine milieu and detect damage-associated molecular patterns (DAMPs) present in the area. This information is then used to provide contextual information to other cells about the environment from which the antigens came and promote escalation of the immune response or antigen tolerance (Wculek 2020).

When considering the implications of stress on dendritic cell function, it is important to note that adrenergic stress has been shown to limit cytokine production from dendritic cells after exposure to lipopolysaccharide (Goyarts 2008), impair migration (Maestroni 2000) and chemotaxis (Maestroni 2003) and drive dendritic cells to preferentially promote differentiation of CD4^+^ T cells into type 17 helper T cells (Th17) rather than the type 1 (Th1) cells (Manni 2011) that are important for anti-tumour immunity. Although these observations did not take place during cancer studies, implications of these results suggest that β-adrenergic signalling may lead to the impairment of T-cell dependent anti-tumour immune responses initiated by dendritic cells (Knochelmann 2018).

During the development of dendritic cells, glucocorticoid exposure has been shown to impair maturation, leading to poor antigen presentation and decreased cytokine production (Piemonti 1999). Rozkova et al (2006) assessed the impact of glucocorticoid exposure during both dendritic cell differentiation and maturation, and discovered further evidence suggesting an immunosuppressive role of glucocorticoid stress, although the magnitude of these findings appears to be culture-condition dependent (Vieira 1998; Canning 2000). Additionally, their ability to activate Th1 cells was shown to be limited, leading to increased development of immunosuppressive regulatory T cells (Tregs) (Matyszak 2000).

Subsequent investigations using a murine breast cancer model determined that, when adrenergic stress is induced by chronic cold stress, dendritic cell populations become more immature and less capable of stimulating CD8^+^ T-cell proliferation (Kokolus 2014). Additional work reported a similar decrease in maturation of antigen-specific dendritic cells when mice were exposed to social disruption stress during treatment with a cancer vaccine to a murine model of melanoma (Sommershof 2017). When Botta & Maestroni used a tumour antigen-specific dendritic cell vaccine strategy to target a thymoma cell line expressing the ovalbumin antigen (EL-4 transfected with OVA), they found that an intradermal injection of the β^2^-AR agonist salbutamol impaired the efficacy of the anti-tumour dendritic cell vaccine treatment (Botta 2008). Interestingly, although blockade of this signalling improved vaccine efficacy when immature dendritic cells were used, if the same procedure was carried out with more mature dendritic cells, the vaccine was ineffective and tumour growth progressed similarly to that in control mice treated with phosphate-buffered saline (Botta 2008).

### T cells

If a dendritic cell is going to stimulate an anti-tumour immune response, it will need to travel out of the tumour microenvironment and into a nearby lymph node to present tumour antigens to other immune cells (Chen 2013). One of the key populations responsible for directing this immune response are the T cells, which can generally be broken down into CD8^+^ and CD4^+^ T cells. CD8^+^ T cells are primarily responsible for the direct killing of targeted cells, whereas CD4^+^ T cells participate in coordinating the immune response. In the lymph node, naive CD4^+^ T cells differentiate into various mature populations, primarily helper T cells (Th) or regulatory T cells (Tregs), guided by input from antigen-presenting dendritic cells (Chen 2013). Tregs are immunosuppressive, whereas helper T-cell populations typically fall into one of two categories: Th1 cells promote the cell-mediated immune response key to anti-tumour activity and Th2 cells promote antibody production and the humoral immune response. Other populations, such as Th9 or Th17 cells, can also develop, but their implications on the anti-tumour immune response are conflicting and less characterised (Yan 2020), especially in the context of chronic stress, and therefore will not be discussed here.

During Th1 responses, cytotoxic CD8^+^ T cells are activated and are known to be a major component of effective anti-tumour immunity (Disis 2010). They exert their effects on tumour cells through mechanisms similar to those used by natural killer cells, but with several key distinctions. Rather than targeting general signs of dysregulation, they only attack cells bearing specific antigens. To do so, they primarily employ two general mechanisms to kill a tumour cell. First, these T cells utilise the same effector molecules, perforin and granzymes, used by natural killer cells to permeabilise the cell’s membrane and induce apoptosis (Harty 2000). Additionally, CD8^+^ T cells can present a membrane-bound protein called Fas ligand (FasL) to the targeted cell, which may be expressing the Fas death receptor, and signalling downstream of the Fas death receptor should then also drive apoptotic pathways within the tumour cell, resulting in its death (La 2009).

Interestingly, when treatments with the β-AR agonist isoprenaline were administered daily to mimic chronic stress conditions, the capability of CD8^+^ T cells to directly kill tumour cells was impaired, along with their ability to produce interferon-gamma (IFN-γ) (Nissen 2018). This led to a decrease in the efficacy of several immune-based therapies targeting a mouse model of lymphoma (Eμ-*Myc* B-cell lymphoma), including anti-PD-1 and anti-4-1BB monoclonal antibodies and a tumour antigen vaccine (Nissen 2018). Similar findings were discovered when a social disruption stress model was used to show that chronic stress impaired the immune response to melanoma vaccine by reducing IFN-γ production and killing of antigen-specific CD8^+^ T cells (Sommershof 2017). Using the B16 model of melanoma and the 4T1 model of mammary carcinoma, Bucsek et al showed that the reduction of β-adrenergic signalling by knocking out the β_2_-AR, treating mice with the β-AR antagonist propranolol or relieving chronic thermal stress with warmer housing conditions resulted in an improvement in anti-PD-1 monoclonal antibody therapy, driven by enhanced CD8^+^ T cell function, as evidenced by increased production of IFN-γ and granzyme B (Bucsek 2017).

For a CD8^+^ T cell to obtain its full anti-tumour potential, dramatic metabolic changes must take place during activation to allow for the increased energy demands required for rapid expansion and production of effector molecules (Siska 2015). Interestingly, when CD8+ T cells were exposed to a pan β-AR agonist (isoproterenol) during differentiation, the necessary metabolic changes, such as increased glycolysis and glucose uptake, were unable to occur (Qiao 2019). This suggests that impaired metabolic reprogramming of CD8+ T cells is another mechanism through which β-adrenetgic signalling promotes immunosuppression (Qiao 2019).

In addition to SNS signalling, glucocorticoid stress has also been shown to have profound effects on T-cell function (Ashwell 2000), as one of many mechanisms attributed to T cells’ capacity to drive immune suppression (Kadmiel 2013). By simply exposing mature T cells to glucocorticoids, Brunetti et al showed that apoptosis could be induced in this population (Brunetti 1995), in addition to effects seen in less differentiated subsets of T cells by other researchers. Such effects include a skewing of the Th1:Th2 ratio towards a Th2 response (Ramirez 1996; Franchimont 2000) and the downregulation of FasL (Yang 1995), which should lead to impaired anti-tumour immunity.

It is also important to appreciate that stress caused by models not specific to HPA or SNS signalling, such as the chronic restraint of mice, leads to an increase in tumour growth driven by a significant reduction in circulating CD4^+^ T cells, a decrease in the production of the key Th1 cytokines IFN-γ and TNF-α, and the impairment of direct lymphocyte cytotoxicity (Frick 2009). Interesting work by Hou et al described another novel model in which repeated exposure to the sound of a scream was used to cause chronic stress. They found that mice exposed to the screaming sound had elevated levels of both corticosterone and noradrenaline, and these mice experienced increased colon cancer growth, increased Th2 cytokine production and decreased Th1 cytokine production (Hou 2013).

Importantly, human clinical trials that aimed to decrease stress through psychosocial telephone counselling similarly found that when patients received counselling during treatment for cervical cancer, they not only had improved measures of quality of life, but also experienced a shift in Th1/Th2 cytokine production towards Th1 (Nelson 2008).

### Regulatory T cells

To constrain and prevent excessive immune responses, regulatory T cells (Tregs) are present throughout the body (Romano 2019). Although Tregs are essential for the prevention of pathological processes such as autoimmunity, their involvement in an anti-tumour immune response decreases cytotoxic T-cell activity, thus promoting tumour growth (Zou 2006). Tregs are CD4^+^ T cells that develop either in the thymus or the periphery and limit cytotoxic T-cell abundance and effector functions through various processes (Romano 2019).

The first of three primary mechanisms by which Tregs limit the immune response is the expression of cytotoxic T-lymphocyte-associated protein 4 (CTLA-4) (Corthay 2009). CTLA-4 is a cell-surface protein that binds a protein (B7) expressed on antigen-presenting cells, thus limiting proper co-stimulation of naive T cells and preventing the maturation of naive T cells into mature cytotoxic T cells (Corthay 2009). Second, Tregs express the receptor CD25, which is capable of binding and sequestering the cytokine interleukin-2 (Chinen 2016). Interleukin-2 is an important cytokine responsible for promoting cytotoxicity of T and natural killer cells and for the proper development and activation of many other immune cells, including CD8^+^ memory T cells, B cells and macrophages (Bachmann 2007). Finally, Tregs produce several immunosuppressive cytokines, such as transforming growth factor beta (TGF-β) and interleukin-10, which are responsible for decreasing T-cell proliferation and inhibiting the production of pro-inflammatory cytokines respectively (Corthay 2009). All of the above functions impair anti-tumour immunity, so changes in the number of Tregs can have dramatic consequences on anti-tumour immunity.

When chronic subordinate-colony housing techniques were used to induce chronic stress in mice, transplanted syngeneic fibrosarcoma tumours grew at a greater rate and an increase in Tregs was discovered (Schmidt 2016). In other studies evaluating the impact of both acoustic stress and chronic restraint stress on a pancreatic mouse model, it was found that not only did stress increase the number of Tregs in the tumour microenvironment, but there it also reduced the production of both Th1 and Th2 cytokines and increased the production of TGF-β (Partecke 2016). In a series of studies evaluating the impact of radical mastectomy surgery on neuroendocrine signalling in humans being treated for primary breast cancer, Zhou et al found that levels of adrenaline and noradrenaline increased after surgery, along with the number and immunosuppressive capacity of Tregs (Zhou 2016). Importantly, this increase in Treg number and function could be abrogated with proprandol treatment (Zhou 2016).

### Myeloid-derived suppressor cells

Myeloid-derived suppressor cells (MDSCs) are a heterogeneous group of myeloid cells that become prevalent and functional during various pathological inflammatory processes, primarily in the context of various cancers. This collection of relatively immature myeloid cells is identifiable in mice and humans on the basis of cell surface markers, and they promote immune suppression through their interactions with other cells in the tumour microenvironment. MDSCs are capable of suppressing the immune system, primarily by acting on T cells, through an abundance of mechanisms, including arginine metabolism by arginase I and inducible nitric oxide synthase (iNOS), reactive oxygen spedes (ROS) production, prostaglandin E_2_ production via cydooxygenase 2 expression (Take 2020), TGF-β and interleukin-10 release, and indoleamine 2,3-dioxygenase synthesis (Groth 2019), and therefore if stress leads to an increase in MDSCs, this is suggestive of impaired anti-tumour immunity.

In addition to the increased Treg development that occurred when mice were exposed to chronic subordinate-colony housing, this form of chronic stress also increased the accumulation of MDSCs, further contributing to the immunosuppressive environment that resulted in increased fibrosarcoma growth (Schmidt 2016). Studies of hepatocellular carcinoma revealed that chronic restraint stress resulted in a redistribution of myeloid cells from the spleen to the tumour, causing increased tumour growth that could be inhibited with propranolol treatment (Jiang 2020). Mohammadpour et al showed that increased β_2_-AR signalling in mice resulted in increased growth of 4T1 and AT-3 mammary carcinomas and an increase in the accumulation and immunosuppressive function of MDSCs (Mohammadpour 2019), and Mundy-Bosse et al found that patients reporting higher levels of stress had increased levels of MDSCs that correlated with increased salivary cortisol levels (Mundy-Bosse 2011).

### Macrophages

Under normal physiological conditions, macrophages are responsible for phagocytosing pathogens and debris, remodelling microenvironments and maintaining tissue homeostasis (Ostuni 2015). However, when macrophages are recruited to the tumour microenvironment, factors such as hypoxia, low pH and cellular debris from dying tumour cells drive tumour-associated macrophages (TAMs) to aid in tumour growth, neovascularisation, metastasis and immunosuppression (Ostuni 2015). Many of the immunosuppressive capabilities of macrophages are similar to those of MDSCs, and this includes production of interleukin-10, TGF-β and prostaglandin E_2_, breakdown of L-arginine by arginase 1 and iNOS, and expression of programmed death ligand 1 (PD-L1) (Ostuni 2015).

Qin et al showed that an increase in adrenergic tone from social isolation of mice resulted in increased M2 macrophage polarisation and increased growth of 4T1 tumours (Qin 2015). Other work studying macrophage recruitment found that elevated adrenergic stress resulted in an increase in monocyte chemotactic factor 1 (MCP1), which led to an increase in macrophage recruitment to the tumour and poor survival (Armaiz-Pena 2015). Additional work studying the impact of adrenergic stress on lung macrophage populations found that stress promoted a pro-tumour macrophage population in the lungs that increased rates of metastasis (Chen 2018) and increased M2 polarisation of macrophages in the tumour microenvironment (Sloan 2010).

## Summary and recommendations

It is clear that there are mixed data in the recent literature related to effects of stress on cancer. Nevertheless, there is strong evidence of a negative effect of stress in some populations, and there is an urgent need to conduct further studies linking perceived stress with measurable and objective physiological alterations produced by the systemic biological effects of that stress.

One emerging area of great interest is the growing body of recent evidence implicating the neuroendocrine stress responses, mediated by the SNS and the HPA axis, as factors capable of impairing anti-tumour immunity. As described above, and in Fig. 2, the function of several generally anti-tumour immune cells, including natural killer cells, T cells and dendritic cells, can be limited by these stress responses, while several immunosuppressive cells, including regulatory T cells, MDSCs and macrophages, become more capable of constraining anti-tumour immunity. As further details of how chronic stress subdues the immune system are discovered, it will be increasingly important to translate these findings to clinical studies. However, to date, nearly all of this research has been generated using preclinical mouse models of cancer. In the future, objective measurements based on immune function could supplement the current standardised questionnaires that are commonly used to assess stress in patients. Without more specific attention being paid to the impact of chronic stress on intermediate factors such as anti-tumour immunity, a full and accurate assessment of the influence of stress on cancer risk and progression in humans could remain elusive.

## Supplementary Material

Supplemental Reference List

Supplemental Material

## LEARNING OBJECTIVES

After reading this article you will be able to:

- understand that there are still considerable uncertainties in determining whether stressful experiences in patients contribute to their cancer progression
- generally describe the key immune cells involved in the anti-tumour immune response
- describe how the major neuroendocrine stress response pathways affect the various cells involved in anti-tumour immunity.

## Appendix

### MCQs

Select the single best option for each question stem

1. **Which of the following immune cell populations are generally considered to be primarily pro-tumour?**
  - CDS^+^ T cells
  - natural killer cells
  - myeloid-derived suppressor cells (MDSCs)
  - a, b and c
  - neither a, b or c, as they all improve the immune response to cancer.
2. **Which of the following is a key molecule that T cells and natural killer cells use to destroy tumour cells?**
  - transforming growth factor beta (TGF-β)
  - vascular endothelial growth factor
  - noradrenaline
  - granzyme B
  - prostaglandin E_2_.
3. **Which of the following immune cells is the key innate immune cell that commonly destroys tumour cells directly?**
  - CD8^+^ T cells
  - macrophages
  - natural killer cells
  - dendritic cells
  - regulatory T cells (Tregs).
4. **Noradrenaline is considered a:**
  - catecholamine
  - β-blocker
  - glucocorticoid
  - mineralocorticoid
  - non-steroidal anti-inflammatory drug.
5. **Which of the following would be the best way to respond to a patient being treated for cancer who describes experiencing psychological stress?**
  - focus on the patient’s physical symptoms, medication and other treatments, because those are the only important aspects of treatment for someone with cancer
  - listen to the patient empathetically and take these findings seriously, because this psychological stress could negatively affect their cancer treatment response
  - listen to the patient, but do not follow-up with them about these complaints, because bringing up their causes of stress will only make things worse
  - listen to the patient, but do not worry about their psychological state because it does not affect the outcome of their cancer treatment
  - listen to the patient and increase their pain medication dose, because that is most likely the cause of their problems.

MCQ answers

1 c 2 d 3 c 4 a 5 b
