# Supplementary material for: Psychosocial stress and immunosuppression in cancer: what can we learn from new research?
Source: BJPsych Adv. Author manuscript; Available in PMC 2021 Jul 21. (PMC8294471; doi:10.1192/bja.2021.9)
Supplement: Supplemental Reference List [file NIHMS1704137-supplement-Supplemental_Reference_List.docx]

Supplemental Reference List:

AHN, H. K., BAE, J. H., AHN, H. Y. & HWANG, I. C. 2016. Risk of cancer among patients with depressive disorder: a meta-analysis and implications. *Psycho-oncology,* 25**,** 1393-1399.

ARCHER, G., PIKHART, H. & HEAD, J. 2015. Do depressive symptoms predict cancer incidence?: 17-year follow-up of the Whitehall II study. *Journal of psychosomatic research,* 79**,** 595-603.

BATTY, G. D., MCINTOSH, A. M., RUSS, T. C., DEARY, I. J. & GALE, C. R. 2016. Psychological distress, neuroticism, and cause-specific mortality: early prospective evidence from UK Biobank. *Journal of epidemiology and community health,* 70**,** 1136-1139.

BATTY, G. D., MORTENSEN, L. H., GALE, C. R., SHIPLEY, M. J., ROBERTS, B. A. & DEARY, I. J. 2009. IQ in late adolescence/early adulthood, risk factors in middle age, and later cancer mortality in men: the Vietnam Experience Study. *Psycho-oncology,* 18**,** 1122-1126.

BATTY, G. D., RUSS, T. C., STAMATAKIS, E. & KIVIMÄKI, M. 2017. Psychological distress in relation to site specific cancer mortality: pooling of unpublished data from 16 prospective cohort studies. *BMJ (Clinical research ed.),* 356**,** j108.

BEHRENDT, C. E., COSGROVE, C. M., JOHNSON, N. J. & ALTEKRUSE, S. F. 2019. Are associations between psychosocial stressors and incident lung cancer attributable to smoking? *PloS one,* 14**,** e0218439.

BLEIKER, E. M. A., HENDRIKS, J. H. C. L., OTTEN, J. D. M., VERBEEK, A. L. M. & VAN DER PLOEG, H. M. 2008. Personality factors and breast cancer risk: a 13-year follow-up. *Journal of the National Cancer Institute,* 100**,** 213-218.

BUTOW, P., PRICE, M., COLL, J., TUCKER, K., MEISER, B., MILNE, R., WILSON, J., HEINIGER, L., BAYLOCK, B., BULLEN, T., WEIDEMAN, P. & PHILLIPS, K.-A. 2018. Does stress increase risk of breast cancer? A 15-year prospective study. *Psycho-oncology,* 27**,** 1908-1914.

CHAPMAN, B. P., FISCELLA, K., KAWACHI, I., DUBERSTEIN, P. & MUENNIG, P. 2013. Emotion suppression and mortality risk over a 12-year follow-up. *Journal of psychosomatic research,* 75**,** 381-385.

CHIRIAC, V.-F., BABAN, A. & DUMITRASCU, D. L. 2018. Psychological stress and breast cancer incidence: a systematic review. *Clujul medical (1957),* 91**,** 18-26.

CLOUSTON, S. A. P., KUAN, P., KOTOV, R., MUKHERJEE, S., THOMPSON-CARINO, P., BROMET, E. J. & LUFT, B. J. 2019. Risk factors for incident prostate cancer in a cohort of world trade center responders. *BMC psychiatry,* 19**,** 389.

FLENSBORG-MADSEN, T., JOHANSEN, C., GRØNBÆK, M. & MORTENSEN, E. L. 2011. A prospective association between quality of life and risk for cancer. *European journal of cancer (Oxford, England : 1990),* 47**,** 2446-2452.

HAMER, M., CHIDA, Y. & MOLLOY, G. J. 2009. Psychological distress and cancer mortality. *Journal of psychosomatic research,* 66**,** 255-258.

HASKINS, C. B., MCDOWELL, B. D., CARNAHAN, R. M., FIEDOROWICZ, J. G., WALLACE, R. B., SMITH, B. J. & CHRISCHILLES, E. A. 2019. Impact of preexisting mental illness on breast cancer endocrine therapy adherence. *Breast cancer research and treatment,* 174**,** 197-208.

JANSEN, F., VERDONCK-DE LEEUW, I. M., CUIJPERS, P., LEEMANS, C. R., WATERBOER, T., PAWLITA, M., PENFOLD, C., THOMAS, S. J., WAYLEN, A. & NESS, A. R. 2018. Depressive symptoms in relation to overall survival in people with head and neck cancer: A longitudinal cohort study. *Psycho-oncology,* 27**,** 2245-2256.

JAYADEVAPPA, R., MALKOWICZ, S. B., CHHATRE, S., JOHNSON, J. C. & GALLO, J. J. 2012. The burden of depression in prostate cancer. *Psycho-oncology,* 21**,** 1338-1345.

JOKELA, M., BATTY, G. D., HINTSA, T., ELOVAINIO, M., HAKULINEN, C. & KIVIMÄKI, M. 2014. Is personality associated with cancer incidence and mortality? An individual-participant meta-analysis of 2156 incident cancer cases among 42,843 men and women. *British journal of cancer,* 110**,** 1820-1824.

KENNEDY, B., VALDIMARSDÓTTIR, U., SUNDSTRÖM, K., SPARÉN, P., LAMBE, M., FALL, K. & FANG, F. 2014. Loss of a parent and the risk of cancer in early life: a nationwide cohort study. *Cancer causes & control : CCC,* 25**,** 499-506.

KNUDSEN, A. K., BERGE, L. I., SKOGEN, J. C., VEDDEGJÆRDE, K.-E. & WILHELMSEN, I. 2015. The prospective association between health anxiety and cancer detection: A cohort study linking the Hordaland Health Study (HUSK) with the Norwegian Cancer Registry. *Journal of Psychosomatic Research,* 79**,** 148-152.

KONDO, N., SAITO, M., HIKICHI, H., AIDA, J., OJIMA, T., KONDO, K. & KAWACHI, I. 2015. Relative deprivation in income and mortality by leading causes among older Japanese men and women: AGES cohort study. *Journal of epidemiology and community health,* 69**,** 680-685.

LEMOGNE, C., CONSOLI, S. M., GEOFFROY-PEREZ, B., COEURET-PELLICER, M., NABI, H., MELCHIOR, M., LIMOSIN, F., ZINS, M., DUCIMETIÈRE, P., GOLDBERG, M. & CORDIER, S. 2013a. Personality and the risk of cancer: a 16-year follow-up study of the GAZEL cohort. *Psychosomatic medicine,* 75**,** 262-271.

LEMOGNE, C., CONSOLI, S. M., MELCHIOR, M., NABI, H., COEURET-PELLICER, M., LIMOSIN, F., GOLDBERG, M. & ZINS, M. 2013b. Depression and the risk of cancer: a 15-year follow-up study of the GAZEL cohort. *American journal of epidemiology,* 178**,** 1712-1720.

LEMOGNE, C., TURINICI, M., PANJO, H., NGO, C., CANOUI-POITRINE, F., CHAUVET-GELINIER, J.-C., LIMOSIN, F., CONSOLI, S. M., GOLDBERG, M., ZINS, M. & RINGA, V. 2018. Personality and breast cancer screening in women of the GAZEL cohort study. *Cancer medicine,* 7**,** 515-524.

LIN, Y., WANG, C., ZHONG, Y., HUANG, X., PENG, L., SHAN, G., WANG, K. & SUN, Q. 2013. Striking life events associated with primary breast cancer susceptibility in women: a meta-analysis study. *Journal of experimental & clinical cancer research : CR,* 32**,** 53.

LIU, B., FLOUD, S., PIRIE, K., GREEN, J., PETO, R. & BERAL, V. 2016. Does happiness itself directly affect mortality? The prospective UK Million Women Study. *Lancet (London, England),* 387**,** 874-881.

MINAMI, Y., HOSOKAWA, T., NAKAYA, N., SUGAWARA, Y., NISHINO, Y., KAKUGAWA, Y., FUKAO, A. & TSUJI, I. 2015. Personality and breast cancer risk and survival: the Miyagi cohort study. *Breast cancer research and treatment,* 150**,** 675-684.

MYINT, P. K., LUBEN, R. N., SURTEES, P. G., WAINWRIGHT, N. W. J., WELCH, A. A., BINGHAM, S. A., WAREHAM, N. J., SMITH, R. D., HARVEY, I. M. & KHAW, K.-T. 2007. Self-Reported Mental Health-Related Quality of Life and Mortality in Men and Women in the European Prospective Investigation into Cancer (EPIC-Norfolk): A Prospective Population Study. *Psychosomatic Medicine,* 69.

NABI, H., KIVIMÄKI, M., ZINS, M., ELOVAINIO, M., CONSOLI, S. M., CORDIER, S., DUCIMETIÈRE, P., GOLDBERG, M. & SINGH-MANOUX, A. 2008. Does personality predict mortality? Results from the GAZEL French prospective cohort study. *International journal of epidemiology,* 37**,** 386-396.

NAKAYA, N. 2014. Effect of psychosocial factors on cancer risk and survival. *Journal of epidemiology,* 24**,** 1-6.

NAKAYA, N., BIDSTRUP, P. E., EPLOV, L. F., SAITO-NAKAYA, K., KURIYAMA, S., TSUJI, I., UCHITOMI, Y. & JOHANSEN, C. 2009. Mental vulnerability and survival after cancer. *Epidemiology (Cambridge, Mass.),* 20**,** 916-920.

NAKAYA, N., BIDSTRUP, P. E., SAITO-NAKAYA, K., FREDERIKSEN, K., KOSKENVUO, M., PUKKALA, E., KAPRIO, J., FLODERUS, B., UCHITOMI, Y. & JOHANSEN, C. 2010. Personality traits and cancer risk and survival based on Finnish and Swedish registry data. *American journal of epidemiology,* 172**,** 377-385.

NAKAYA, N., SAITO-NAKAYA, K., AKECHI, T., KURIYAMA, S., INAGAKI, M., KIKUCHI, N., NAGAI, K., TSUGANE, S., NISHIWAKI, Y., TSUJI, I. & UCHITOMI, Y. 2008. Negative psychological aspects and survival in lung cancer patients. *Psycho-oncology,* 17**,** 466-473.

NIEDZWIEDZ, C. L., ROBB, K. A., KATIKIREDDI, S. V., PELL, J. P. & SMITH, D. J. 2020. Depressive symptoms, neuroticism, and participation in breast and cervical cancer screening: Cross-sectional and prospective evidence from UK Biobank. *Psycho-oncology,* 29**,** 381-388.

NIELSEN, N. R., KRISTENSEN, T. S., SCHNOHR, P. & GRØNBAEK, M. 2008. Perceived stress and cause-specific mortality among men and women: results from a prospective cohort study. *American journal of epidemiology,* 168**,** 481-491.

NIELSEN, N. R., STRANDBERG-LARSEN, K., GRØNBAEK, M., KRISTENSEN, T. S., SCHNOHR, P. & ZHANG, Z.-F. 2007. Self-reported stress and risk of endometrial cancer: a prospective cohort study. *Psychosomatic medicine,* 69**,** 383-389.

PINQUART, M. & DUBERSTEIN, P. R. 2010. Depression and cancer mortality: a meta-analysis. *Psychological medicine,* 40**,** 1797-1810.

RANCHOR, A. V., SANDERMAN, R. & COYNE, J. C. 2010. Invited commentary: personality as a causal factor in cancer risk and mortality--time to retire a hypothesis? *American journal of epidemiology,* 172**,** 386-388.

SAWADA, T., NISHIYAMA, T., KIKUCHI, N., WANG, C., LIN, Y., MORI, M., TANNO, K., TAMAKOSHI, A. & KIKUCHI, S. 2016. The influence of personality and perceived stress on the development of breast cancer: 20-year follow-up of 29,098 Japanese women. *Scientific reports,* 6**,** 32559.

SCHOORMANS, D., HUSSON, O., DENOLLET, J. & MOLS, F. 2017. Is Type D personality a risk factor for all-cause mortality? A prospective population-based study among 2625 colorectal cancer survivors from the PROFILES registry. *Journal of psychosomatic research,* 96**,** 76-83.

SVENSSON, T., INOUE, M., SAWADA, N., CHARVAT, H., IWASAKI, M., SASAZUKI, S., SHIMAZU, T., YAMAJI, T., KAWAMURA, N., SHIBUYA, K., MIMURA, M. & TSUGANE, S. 2016. Coping strategies and cancer incidence and mortality: The Japan Public Health Center-based prospective study. *Cancer epidemiology,* 40**,** 126-133.

TRUDEL-FITZGERALD, C., POOLE, E. M., IDAHL, A., LUNDIN, E., SOOD, A. K., KAWACHI, I., KUBZANSKY, L. D. & TWOROGER, S. S. 2017. The Association of Work Characteristics With Ovarian Cancer Risk and Mortality. *Psychosomatic medicine,* 79**,** 1059-1067.

TRUDEL-FITZGERALD, C., POOLE, E. M., SOOD, A. K., OKEREKE, O. I., KAWACHI, I., KUBZANSKY, L. D. & TWOROGER, S. S. 2019. Social Integration, Marital Status, and Ovarian Cancer Risk: A 20-Year Prospective Cohort Study. *Psychosomatic medicine,* 81**,** 833-840.

WAKAI, K., KOJIMA, M., NISHIO, K., SUZUKI, S., NIWA, Y., LIN, Y., KONDO, T., YATSUYA, H., TAMAKOSHI, K., YAMAMOTO, A., TOKUDOME, S., TOYOSHIMA, H. & TAMAKOSHI, A. 2007. Psychological attitudes and risk of breast cancer in Japan: a prospective study. *Cancer causes & control : CCC,* 18**,** 259-267.

WHITE, V. M., ENGLISH, D. R., COATES, H., LAGERLUND, M., BORLAND, R. & GILES, G. G. 2007. Is cancer risk associated with anger control and negative affect? Findings from a prospective cohort study. *Psychosomatic medicine,* 69**,** 667-674.

WILTINK, J., BEUTEL, M. E., TILL, Y., OJEDA, F. M., WILD, P. S., MÜNZEL, T., BLANKENBERG, S. & MICHAL, M. 2011. Prevalence of distress, comorbid conditions and well being in the general population. *Journal of affective disorders,* 130**,** 429-437.

WU, Y., AI, Z. & XU, G. 2017. Marital status and survival in patients with non-small cell lung cancer: an analysis of 70006 patients in the SEER database. *Oncotarget,* 8**,** 103518-103534.

ZVOLENSKY, M. J., TAHA, F., BONO, A. & GOODWIN, R. D. 2015. Big five personality factors and cigarette smoking: a 10-year study among US adults. *Journal of psychiatric research,* 63**,** 91-96.
