# Supplementary material for: Psychosocial stress and immunosuppression in cancer: what can we learn from new research?
Source: BJPsych Adv. Author manuscript; Available in PMC 2021 Jul 21. (PMC8294471; doi:10.1192/bja.2021.9)
Supplement: Supplementary file 2 [file NIHMS1704137-supplement-Supplemental_Material.docx]

Incidence

Breast Cancer

Null	Factor Tested	Ratio Type	Ratio	Low CI	High CI
White et al. 2007					
	Anger Control	HR	1	0.9	1.11
	Negative Affect	HR	0.98	0.88	1.08
Bleiker et. al 2008					
	High emotional control and low	OR	1.04	0.59	1.83
	Low emotional expression-out and low social support	OR	1.14	0.77	1.68
	High anxiety and high emotional expression – in	OR	1	0.65	1.59
	High depression and low optimism	OR	0.75	0.52	1.07
	High anger and high antiemotionality	OR	0.74	0.4	1.36
	High anger and high understanding	OR	1.97	0.98	4
	High anger and high emotional control	OR	0.78	0.37	1.65
	Type C Personality	OR	0.85	0.62	1.17
Lemogne et al. 2013					
	Depression - CES-D > Threshold	HR	1.01	0.66	1.55
Jokela et al. 2014					
	Extraversion	OR	1	0.87	1.15
	Neuroticism	OR	1.06	0.8	1.4
	Agreeableness	OR	0.96	0.83	1.11
	Conscientiousness	OR	1.11	0.97	1.27
	Openness to Experience	OR	1.04	0.92	1.19
Minami et al. 2015					
	Extraversion ≤ 2	HR	1	1	1
	Extraversion 3-4	HR	0.84	0.56	1.28
	Extraversion 5-7	HR	0.99	0.68	1.44
	Extraversion 8≤	HR	0.92	0.62	1.36
	Neuroticism ≤ 2	HR	1	1	1
	Neuroticism 3-4	HR	1.02	0.69	1.49
	Neuroticism 5-7	HR	1.03	0.7	1.5
	Neuroticism 8≤	HR	0.96	0.67	1.37
	Psychoticism ≤ 2	HR	1	1	1
	Psychoticisim 3-4	HR	0.9	0.59	1.39
	Psychoticism 5-7	HR	1.07	0.7	1.63
	Psychoticism 8≤	HR	1.04	0.7	1.56
	Lie ≤ 2	HR	1	1	1
	Lie 3-4	HR	0.89	0.61	1.3
	Lie 5-7	HR	0.82	0.56	1.2
	Lie 8≤	HR	0.84	0.55	1.29
Archer et al. 2015					
	Chronic Depressive Symptoms	HR	1.09	0.69	1.73
	New Depressive Symptoms < 9 yrs Follow up	HR*	1.89	1.23	2.9
	New Depressive Symptoms ≥ 9 yrs	HR	0.84	0.52	1.35
[illegible]					

Incidence

Breast Cancer

Null	Factor Tested	Ratio Type	Ratio	Low CI	High CI
Sawada et al. 2016					
	"Ikigai" - Agree	HR	0.77	0.43	1.37
	"Ikigai" - Strongly Agree	HR	0.81	0.41	1.62
	Decisiveness - Agree	HR	1.07	0.62	1.85
	Easily Angered - Agree	HR	0.98	0.55	1.76
	Perceived Stress - Agree	HR	1.71	1.02	2.85
	Perceived Stress - Agree Strongly	HR	1	0.56	1.78
Ahn et al. 2016					
	Depressive Disorder	OR	1.42	0.94	2.13
Lemogne et al. 2018					
	Type A Personality - Not including Depressive Symptoms	OR	1.5	1.16	2
	Type A Personality - Including Depressive Symptoms	OR	1.46	1.13	1.9
Butow et al. 2018					
	Total Acute Stressors	HR	1.03	0.99	1.08
	Total Chronic Stressors	HR	1	0.9	1.11

Incidence

Breast Cancer

Harmful	Factor Tested	Ratio Type	Ratio	Low CI	High CI
Wakai et al. 2007					
	Type A	RR	0.56	0.36	0.87
	Hostile	RR	0.85	0.51	0.87
	Stress Prone	RR	1.01	0.69	1.5
	"Ikigai"	RR	0.66	0.47	0.94
Nakaya et al. 2010					
	Extraversion	HR	0.97	0.95	1
	Neuroticism	HR	1.03	1	1.05
Lemogne et al. 2013					
	Type 1	HR	0.81	0.67	0.97
	Type 5	HR	0.97	0.81	1.15
	Hostility	HR	0.88	0.73	1.07
	Type A	HR	1.05	0.87	1.27
Lin et al. 2013					
	Striking Life Event	OR	1.51	1.15	1.97
	Severe Striking Life Event	OR	2.07	1.06	4.03
Svensson et al. 2016					
	Avoidance Behavior	HR	1.91	1.26	2.91
	Combined Coping Strategy	HR	2.32	1.15	4.7
Batty et al. 2017					
	Psychological Distress - High vs Low	HR	1.27	0.72	2.23
Chiriac et al. 2018					
Niedzwiedz et al. 2019					
	Severe Depressive Symptoms & Screening	OR	0.96	0.95	0.97
	Neuroticism & Screening	OR	1.009	1.002	1.016

Mortality

Breast Cancer

Harmful	Factor Tested	Ratio Type	Ratio	Low CI	High CI
Pinquart et al. 2010					
	Depression	HR	1.18	1.07	1.29
Svensson et al. 2016					
	Combined Coping Strategy	HR	9.9	2.24	43.81
Batty et al. 2017					
	Psychological Distress - High vs Low	HR	1.91	1.2	3.05
**Null**	**Factor Tested**	**Ratio Type**	**Ratio**	**Low CI**	**High CI**
Nakaya et al. 2010					
	Extraversion	HR	0.98	0.92	1.04
	Neuroticism	HR	1.02	0.95	1.09
Minami et al. 2015					
	Extraversion ≤ 2	HR	1	1	1
	Extraversion 3-4	HR	0.42	0.13	1.4
	Extraversion 5-7	HR	0.38	0.13	1.1
	Extraversion 8≤	HR	0.43	0.15	1.28
	Neuroticism ≤ 2	HR	1	1	1
	Neuroticism 3-4	HR	1.08	0.33	3.59
	Neuroticism 5-7	HR	1.13	0.34	3.74
	Neuroticism 8≤	HR	1.22	0.42	3.57
	Psychoticism ≤ 2	HR	1	1	1
	Psychoticisim 3-4	HR	0.58	0.13	2.58
	Psychoticism 5-7	HR	1.19	0.38	3.72
	Psychoticism 8≤	HR	0.67	0.19	2.37
	Lie ≤ 2	HR	1	1	1
	Lie 3-4	HR	1.04	0.33	3.28
	Lie 5-7	HR	0.69	0.24	2.02
	Lie 8≤	HR	0.48	0.15	1.54

Survival

Breast Cancer

Harmful	Factor Tested	Ratio Type	Ratio	Low CI	High CI
Haskins et al. 2019					
	Any Mental Illness - Treatment Initiation	HR	0.95	0.92	0.98
	Any Mental Illness - Treatment Discontinuation	HR	1.19	1.11	1.27
	Any Mental Illness - Treatment Adherence	HR	0.006	-0.012	0

Incidence

Prostate Cancer

Harmful	Factor Tested	Ratio Type	Ratio	Low CI	High CI
Lemogne et al. 2013					
	Type 1	HR	1.01	0.91	1.12
	Type 5	HR	1.02	0.91	1.13
	Hostility	HR	1.08	0.97	1.21
	Type A	HR	1.02	0.92	1.14
Ahn et al. 2016					
	Depressive Disorder	OR	1.22	0.62	2.4
Clouston et al. 2019					
	Severity of Re-experiencing Stressful	HR	3.5	1.37	8.96
	World Trade Center Exposure Severity	HR	0.99	0.86	1.14
**Null**	**Factor Tested**	**Ratio Type**	**Ratio**	**Low CI**	**High CI**
White et al. 2007					
	Anger Control	HR	1.17	1.04	1.3
	Negative Affect	HR	1	0.89	1.13
Nakaya et al. 2010					
	Extraversion	HR	1	0.96	1.04
	Neuroticism	HR	0.96	0.92	1
Lemogne et al. 2013					
	Depression - CES-D > Threshold	HR	1.07	0.84	1.38
Jokela et al. 2014					
	Extraversion	OR	1.05	0.91	1.21
	Neuroticism	OR	1.04	0.89	1.21
	Agreeableness	OR	1.01	0.88	1.16
	Conscientiousness	OR	0.87	0.75	1.01
	Openness to Experience	OR	1.12	0.98	1.29

Mortality

Prostate Cancer

Harmful	Factor Tested	Ratio Type	Ratio	Low CI	High CI
Jayadevappa et al. 2012					
	Treatment Phase Depression -	HR	2.82	2.6	3.06
	Post Treatment Depression - Unadjusted	HR	0.86	0.79	0.92
	Treatment Phase Depression - Adjusted for Clinical and Demographic Covariates	HR	2.01	1.85	2.18
	Post Treatment Depression - Adjusted	HR	0.75	0.7	0.81
	Treatment Phase Depression - Adjusted for Propensity Score	HR	2.09	1.92	2.27
	Post Treatment Depression - Adjusted for Propensity Score	HR	0.76	0.71	0.8
	Treatment Phase Depression - Instrument Variable Adjusted	HR	2.06	1.96	2.16
	Post Treatment Depression - Instrument Variable Adjusted	HR	0.76	0.73	0.79
Batty et al. 2017					
	Psychological Distress - High vs Low	HR	2.42	1.29	4.54
**Null**	**Factor Tested**	**Ratio Type**	**Ratio**	**Low CI**	**High CI**
Nakaya et al. 2010					
	Extraversion	HR	1.02	0.96	1.09
	Neuroticism	HR	1	0.94	1.07

Incidence

Lung Cancer

Harmful	Factor Tested	Ratio Type	Ratio	Low CI	High CI
Batty et al. 2017					
	Psychological Distress - High vs Low	HR	1.57	0.87	2.84
**Null**	**Factor Tested**	**Ratio Type**	**Ratio**	**Low CI**	**High CI**
White et al. 2007					
	Anger Control	HR	1.19	0.97	1.46
	Negative Affect	HR	1.24	1.01	1.52
Nakaya et al. 2010					
	Extraversion	HR	1.06	1.01	1.1
	Neuroticism	HR	1.05	1.01	1.1
Jokela et al. 2014					
	Extraversion	OR	1.01	0.75	1.37
	Neuroticism	OR	1.32	0.98	1.77
	Agreeableness	OR	0.92	0.6	1.4
	Conscientiousness	OR	1.07	0.79	1.45
	Openness to Experience	OR	0.82	0.62	1.08
Ahn et al. 2016					
	Depressive Disorder	OR	1.47	1.26	1.72
Behrendt et al. 2019					
	Divorced - Male	HR	1.07	0.73	1.58
	Divorced - Female	HR	0.91	0.69	1.19
	Widowed - Male	HR	0.79	0.56	1.1
	Widowed - Female	HR	0.75	0.54	1.04
	Unemployed - Male	HR	1.66	0.89	3.08
	Unemployed - Female	HR	0.55	0.14	2.15

Smoking/Tobacco Related Cancers

Harmful	Factor Tested	Ratio Type	Ratio	Low CI	High CI
Lemogne et al. 2013					
	Type 1	HR	1.13	0.9	1.41
	Type 5	HR	1	0.8	1.25
	Hostility	HR	1.15	0.92	1.44
	Type A	HR	0.96	0.77	1.2
**Null**	**Factor Tested**	**Ratio Type**	**Ratio**	**Low CI**	**High CI**
Lemogne et al. 2013					
	Depression - CES-D > Threshold	HR	0.91	0.59	1.42

Survival

Lung Cancer

Harmful	Factor Tested	Ratio Type	Ratio	Low CI	High CI
Saito-Nakaya et al. 2008					
	Male - Married	HR	1		0
	Male - Widowed	HR	1.7	1.2	2.5
	Male - Separated/Divorced	HR	1.1	0.7	1.7
	Male - Single	HR	0.9	0.5	1.5
	Female - Married	HR	1		0
	Female - Widowed	HR	0.7	0.5	1.1
	Female - Separated/Divorced	HR	0.5	0.3	1.1
	Female - Single	HR	1.2	0.5	2.7
	Total - Married	HR	1		0
	Total - Widowed	HR	1.1	0.9	1.5
	Total - Separated/Divorced	HR	0.9	0.6	1.3
	Total - Single	HR	0.9	0.6	1.4

Mortality

Lung Cancer

Harmful	Factor Tested	Ratio Type	Ratio	Low CI	High CI
Hamer et al. 2009					
	Psychological Distress - Subjects without Cancer History	HR	1.46	0.96	2.22
Pinquart et al. 2010					
	Depression	HR	1.17	1.03	1.34
Batty et al. 2017					
	Psychological Distress - High vs Low	HR	1.26	0.97	1.62
**Null**	**Factor Tested**	**Ratio Type**	**Ratio**	**Low CI**	**High CI**
Nakaya et al. 2008					
	Neuroticism (EPQ-R) ≤ 3	HR	1		0
	Neuroticism (EPQ-R) = 4 - 5	HR	1.1	0.9	1.3
	Neuroticism (EPQ-R) = 6 - 7	HR	1.1	0.9	1.4
	Neuroticism (EPQ-R) ≥ 8	HR	1.2	0.9	1.4
	Helplessness/hopelessness (MACS) ≤ 7	HR	1		0
	Helplessness/hopelessness (MACS) = 8 - 10	HR	0.9	0.7	1.1
	Helplessness/hopelessness (MACS) = 11 - 13	HR	0.9	0.7	1.2
	Helplessness/hopelessness (MACS) ≥ 14	HR	1	0.8	1.3
	Depression (HADS) ≤ 2	HR	1		0
	Depression (HADS) = 3 - 5	HR	1	0.8	1.2
	Depression (HADS) = 6 - 8	HR	1	0.8	1.3
	Depression (HADS) ≥ 9	HR	1.2	0.9	1.4
Nakaya et al. 2010					
	Extraversion	HR	1.05	1	1.11
	Neuroticism	HR	0.96	0.91	1.01
Kondo et al. 2015					
	Relative Deprivation of Income	HR	1.01	0.83	1.23

Smoking/Tobacco Related Cancers

Protective	Factor Tested	Ratio Type	Ratio	Low CI	High CI
Batty et al. 2009					
	IQ < 90 - Adjusted for Age and All Mediating Variables	HR	1.62	0.85	3.11
	90 < IQ < 102 - Adjusted for Age and All Mediating Variables	HR	1.07	0.56	2.05
	102 < IQ < 112 - Adjusted for Age and All Mediating Variables	HR	0.98	0.52	1.85
	IQ ≥ 112 - Adjusted for Age and All Mediating Variables	HR	1	0	0
	Per 1 SD Decrease in IQ - Adjusted for Age and All Mediating Variables	HR	1.22	0.97	1.53

Incidence

Any Cancer

Harmful	Factor Tested	Ratio Type	Ratio	Low CI	High CI
Flensborg-Madsen et al. 2011					
	Poor Quality of Life	HR	1.9	1.1	3.4
	Good Quality of Life	HR	1.31	0.8	2.2
Lemogne et al. 2013					
	Type 1	HR	0.97	0.91	1.04
	Type 5	HR	1.06	0.99	1.13
	Hostility	HR	1.05	0.98	1.12
	Type A	HR	1.01	0.95	1.08
Svensson et al. 2016					
	Approach Oriented Coping Strategy	HR	0.85	0.72	0.99
	Positive Reappraisal	HR	0.84	0.72	0.97
	Approach Oriented Coping Strategy	HR	1.13	1.01	1.27
	Avoidance Oriented Coping Strategy	HR	0.69	0.5	0.94
Batty et al. 2017					
	Psychological Distress - High vs Low	HR	1.16	0.9	1.49
**Null**	**Factor Tested**	**Ratio Type**	**Ratio**	**Low CI**	**High CI**
White et al. 2007					
	Anger Control	HR	1.01	0.96	1.06
	Negative Affect	HR	1.02	0.98	1.07
Nakaya et al. 2010					
	Extraversion	HR	0.99	0.98	1.01
	Neuroticism	HR	1	0.99	1.02
Ranchor et al. 2010					
Nakaya 2014					
	EPI - Extraversion - Sweedish/Finish Cohort	HR	0.99	0.98	1.01
	EPI - Neuroticism - Sweedish/Finish Cohort	HR	1	0.99	1.02
	EPQ-R - High Extraversion - Miyagi Cohort	HR	0.9	0.7	1.1
	EPQ-R - High Neuroticism - Miyagi Cohort	HR	1.2	1	1.4
Jokela et al. 2014					
	Extraversion	OR	1.02	0.97	1.06
	Neuroticism	OR	1.02	0.96	1.08
	Agreeableness	OR	0.98	0.91	1.05
	Conscientiousness	OR	1	0.93	1.07
	Openness to Experience	OR	1.01	0.97	1.05
Knudsen et al. 2015					
	Health Anxiety - Men	HR	1.76	1.06	2.91
	Health Anxiety - Women	HR	1.12	0.69	1.81
Ahn et al. 2016					
	Depressive Disorder	OR	1.26	1.06	1.5

Mortality

Any Cancer

Harmful	Factor Tested	Ratio Type	Ratio	Low CI	High CI
Myint et al. 2007					
	Per 10-point Decrease in Mental Health Component Scores				
Hamer et al. 2009					
	Psychological Distress - All Subjects	HR	1.27	1	1.62
	without Cancer History	HR	1.15	0.88	1.49
	Psychological Distress - Subjects with Cancer History	HR	1.97	1.05	3.71
Pinquart et al. 2010					
	Depression - Early Cancer Stage (I & II)	HR	1.31	1.1	1.57
	Depression - Unspecified Cancer Stage	HR	1.16	1.1	1.21
	Depression - Late Cancer Stage (III & IV)	HR	1.16	1.04	1.29
Chapman et al. 2013					
	Emotion Suppresion	HR	1.7	1	2.88
Batty et al. 2017					
	Psychological Distress - High vs Low	HR	1.26	1.11	1.42
Schoormans et al. 2017					
	Social Inhibition	HR	1.3	0.9	1.8
	Negatice Affectivity	HR	2	1.4	2.8
	Type D Personality	HR	1.7	1.3	2.4
**Null**	**Factor Tested**	**Ratio Type**	**Ratio**	**Low CI**	**High CI**
Nabi et al. 2005					
	Type 1 - Cancer-prone Personality	RII	0.86	0.53	1.41
Nielsen et al. 2008					
	Perceived Stress - Men - Low	HR	1	0	0
	Perceived Stress - Men - Medium	HR	0.95	0.82	1.1
	Perceived Stress - Men - High	HR	1.13	0.86	1.49
	Perceived Stress - Women - Low	HR	1	0	0
	Perceived Stress - Women - Medium	HR	0.94	0.81	1.08
	Perceived Stress - Women - High	HR	0.75	0.58	0.96
Nakaya N et al. 2009					
	Mental Vulnerability - Q1 (Lowest)	HR	1	reference	0
	Mental Vulnerability - Q2	HR	1.1	0.8	1.5
	Mental Vulnerability - Q3	HR	0.9	0.6	1.4
	Mental Vulnerability - Q4 (Highest)	HR	1.1	0.8	1.5
	Mental Vulnerability - Psychosomatic Symptoms ≥ 1	HR	1.2	0.9	1.5
	Mental Vulnerability - Interpersonal Problems ≥ 1	HR	0.9	0.7	1.2
	Mental Vulnerability - Psychological Symptoms ≥ 1	HR	1	0.7	1.3
Nakaya N et al. 2010					
	Extraversion	HR	1	0.98	1.02
	Neuroticism	HR	1	0.98	1.02
Wiltink et al. 2011					
	Depression	HR	1.11	0.7	1.77
	Social Anxiety	HR	1.28	0.83	1.99
	Panic	HR	1.35	0.83	2.2
	Generalized Anxiety	HR	0.98	0.51	1.86
	Type D Personality	HR	0.98	0.75	1.29

Mortality					
**Any Cancer**					
Nakaya 2014					
	EPI - Extraversion - Sweedish/Finish Cohort	HR	1	0.98	1.02
	EPI - Neuroticism - Sweedish/Finish Cohort	HR	1	0.98	1.02
	EPQ-R - High Extraversion - Miyagi Cohort	HR	0.9	0.7	1.1
	EPQ-R - High Neuroticism - Miyagi Cohort	HR	1.2	1	1.4
Jokela et al. 2014					
	Extraversion	OR	1.06	0.88	1.28
	Neuroticism	OR	1.07	0.96	1.19
	Agreeableness	OR	0.94	0.82	1.09
	Conscientiousness	OR	0.94	0.8	1.09
	Openness to Experience	OR	1.09	0.95	1.26
Kondo et al. 2015					
	Relative Deprivation of Income	HR	1.09	0.99	1.21
	Relative Deprivation of Income - Women	HR	1.1	0.94	1.29
	Relative Deprivation of Income - Adjusted for Socioeconomic Status	HR	0.94	0.74	1.19
	Relative Deprivation of Income - Men defined by Gender, age group, and municipality of residence	HR	0.89	0.7	1.12
	Relative Deprivation of Income - Women defined by Gender, age group, and municipality of residence	HR	0.97	0.66	1.44
Liu et al. 2016					
	Unhappiness - Adjusted for Self Reported Health	HR	0.98	0.93	1.02
Batty et al. 2016					
	Distress Categories - 1	HR	1	0	0
	Distress Categories - 2	HR	1.16	1.07	1.28
	Distress Categories - 3	HR	1.54	1.37	1.72
	Distress Categories Based on SD	HR	1.15	1.1	1.2
**Protective**	**Factor Tested**	**Ratio Type**	**Ratio**	**Low CI**	**High CI**
Batty et al. 2009					
	IQ < 90 - Adjusted for Age and All Mediating Variables	HR	1.39	0.84	2.3
	90 < IQ < 102 - Adjusted for Age and All Mediating Variables	HR	1.01	0.62	1.66
	102 < IQ < 112 - Adjusted for Age and All Mediating Variables	HR	0.91	0.56	1.47
	IQ ≥ 112 - Adjusted for Age and All Mediating Variables	HR	1	0	0
	Per 1 SD Decrease in IQ - Adjusted for Age and All Mediating Variables	HR	1.16	0.97	1.39

Incidence

Ovarian Cancer

Harmful	Factor Tested	Ratio Type	Ratio	Low CI	High CI
Niedzwiedz et al. 2019					
	Severe Depressive Symptoms &	OR	0.958	0.95	0.966
	Neuroticism & Screening	OR	0.978	0.973	0.982
Trudel-Fitzgerald et al. 2019					
	Socially Isolated vs Integrated	HR	1.54	1.09	2.19
	Widowed vs Married	HR	1.65	1.21	2.25
**Null**	**Factor Tested**	**Ratio Type**	**Ratio**	**Low CI**	**High CI**
Trudel-Fitzgerald et al. 2017					
	Job Strain - Passive	RR	1.16	0.37	3.6
	Job Strain - Active	RR	1.27	0.87	1.84
	Job Strain - High Strain	RR	1.06	0.72	1.55
	Job Control - Moderate	RR	0.62	0.13	2.92
	Job Control - Low	RR	1.27	0.92	1.76
	Job Demand - Moderate	RR	0.66	0.49	0.9
	Job Demand - High	RR	0.89	0.44	1.77

Cervical Cancer

Harmful	Factor Tested	Ratio Type	Ratio	Low CI	High CI
Niedzwiedz et al. 2019					
	Severe Depressive Symptoms & Screening	OR	0.958	0.95	0.966
	Neuroticism & Screening	OR	0.978	0.973	0.982
**Null**	**Factor Tested**	**Ratio Type**	**Ratio**	**Low CI**	**High CI**
Nakaya et al. 2010					
	Extraversion	HR	0.99	0.91	1.07
	Neuroticism	HR	1.03	0.95	1.11

Endometrial Cancer

Protective	Factor Tested	Ratio Type	Ratio	Low CI	High CI
Nielsen et al. 2007					
	Stress Intensity - Light	HR	0.71	0.42	1.21
	Stress Intensity - Moderate	HR	0.52	0.26	1.04
	Stress Intensity - High	HR	0.72	0.28	1.89
	Stress Frequency - Monthly	HR	0.77	0.44	1.34
	Stress Frequency - Weekly	HR	0.73	0.38	1.42
	Stress Frequency - Daily	HR	0.4	0.14	1.15

Incidence

Colorectal Cancer

Harmful	Factor Tested	Ratio Type	Ratio	Low CI	High CI
Lemogne et al. 2013					
	Type 1	HR	0.91	0.74	1.12
	Type 5	HR	1.11	0.9	1.37
	Hostility	HR	0.89	0.72	1.1
	Type A	HR	0.91	0.74	1.11
Svensson et al. 2016					
	Approach Oriented Coping Strategy	HR	0.64	0.42	0.98
Batty et al. 2017					
	Psychological Distress - High vs Low	HR	1.17	0.58	2.38
**Null**	**Factor Tested**	**Ratio Type**	**Ratio**	**Low CI**	**High CI**
White et al. 2007					
	Anger Control	HR	1.07	0.95	1.2
	Negative Affect	HR	1.14	1.01	1.28
Nakaya et al. 2010					
	Extraversion	HR	0.98	0.94	1.03
	Neuroticism	HR	0.97	0.94	1.01
Lemogne et al. 2013					
	Depression - CES-D > Threshold	HR	0.71	0.43	1.17
Jokela et al. 2014					
	Extraversion	OR	1.09	0.85	1.39
	Neuroticism	OR	0.88	0.65	1.2
	Agreeableness	OR	0.98	0.67	1.44
	Conscientiousness	OR	0.91	0.71	1.15
	Openness to Experience	OR	0.81	0.66	1
Ahn et al. 2016					
	Depressive Disorder	OR	1.28	0.89	1.84

Mortality

Ovarian Cancer

Harmful	Factor Tested	Ratio Type	Ratio	Low CI	High CI
Batty et al. 2017					
	Psychological Distress - High vs Low	HR	2.37	1.24	4.54
**Null**	**Factor Tested**	**Ratio Type**	**Ratio**	**Low CI**	**High CI**
Trudel-Fitzgerald et al. 2017					
	Job Strain - Passive	RR	0.69	0.41	1.17
	Job Strain - Active	RR	0.83	0.5	1.37
	Job Strain - High Strain	RR	1.08	0.64	1.82
	Job Control - Moderate	RR	1.11	0.58	2.1
	Job Control - Low	RR	0.95	0.65	1.4
	Job Demand - Moderate	RR	1.34	0.85	2.11
	Job Demand - High	RR	1.22	0.84	1.77

Mortality

Colorectal Cancer

Harmful	Factor Tested	Ratio Type	Ratio	Low CI	High CI
Batty et al. 2017					
	Psychological Distress - High vs Low	HR	1.84	1.21	2.78
**Null**	**Factor Tested**	**Ratio Type**	**Ratio**	**Low CI**	**High CI**
Nakaya et al. 2010					
	Extraversion	HR	0.97	0.9	1.05
	Neuroticism	HR	0.93	0.86	1.01

Incidence

Harmful	Factor Tested	Ratio Type	Ratio	Low CI	High CI
Kennedy et al. 2014					
HPV Related Cancers	Loss of Parent During Childhood	RR	1.4	1.2	1.7
Pancreatic Cancer	Loss of Parent	RR	2.6	1.9	3.7
Svensson et al. 2016					
Localized Cancer	Approach Oriented Coping Strategy	HR	1.13	1.01	1.27
Screening Detected Cancer	Approach Oriented Coping	HR	1.35	1.15	1.58
Screening Detected Cancer	Planning Behavior	HR	1.27	1.09	1.48
Non-Localized Cancers	Combined Coping Strategy	HR	0.53	0.32	0.88
Non-Screening Detected Cancer	Combined Coping Strategy	HR	0.54	0.36	0.79
**Null**	**Factor Tested**	**Ratio Type**	**Ratio**	**Low CI**	**High CI**
Ahn et al. 2016					
Oral Cancer	Depressive Disorder	OR	1.75	1.22	2.51
Liver Cancer	Depressive Disorder	OR	1.22	0.8	1.87
White et al. 2007					
Melanoma	Anger Control	HR	0.96	0.85	1.09
Melanoma	Negative Affect	HR	1	0.88	1.14
Lemogne et al. 2013					
Lymphoid & Hematopoietic Cancers	Depression - CES-D > Threshold	HR	0.89	0.52	1.52
Other Cancers	Depression - CES-D > Threshold	HR	0.8	0.57	1.12
Jokela et al. 2014					
Leukaemia/Lymphoma	Extraversion	OR	1.01	0.66	1.54
Skin Cancer	Extraversion	OR	0.97	0.81	1.16
Leukaemia/Lymphoma	Neuroticism	OR	1.11	0.85	1.45
Skin Cancer	Neuroticism	OR	0.8	0.58	1.08
Leukaemia/Lymphoma	Agreeableness	OR	0.88	0.67	1.15
Skin Cancer	Agreeableness	OR	0.97	0.73	1.3
Leukaemia/Lymphoma	Conscientiousness	OR	0.95	0.73	1.23
Skin Cancer	Conscientiousness	OR	1.13	0.88	1.44
Leukaemia/Lymphoma	Openness to Experience	OR	1.11	0.85	1.45
Skin Cancer	Openness to Experience	OR	0.94	0.78	1.14
Nakaya et al. 2010					
Stomach Cancer	Extraversion	HR	1.01	0.95	1.07
Liver Cancer	Extraversion	HR	0.98	0.91	1.06
Pancreatic Cancer	Extraversion	HR	0.97	0.9	1.04
Kidney Cancer	Extraversion	HR	0.94	0.87	1.02
Melanoma	Extraversion	HR	1.01	0.95	1.08
Nervous System Cancers	Extraversion	HR	0.98	0.92	1.03
Stomach Cancer	Neuroticism	HR	0.99	0.94	1.05
Liver Cancer	Neuroticism	HR	0.91	0.85	0.98
Pancreatic Cancer	Neuroticism	HR	0.98	0.92	1.05
Kidney Cancer	Neuroticism	HR	0.99	0.92	1.06
Melanoma	Neuroticism	HR	0.99	0.92	1.05
Nervous System Cancers	Neuroticism	HR	1.04	0.99	1.1

Mortality

Harmful	Factor Tested	Ratio Type	Ratio	Low CI	High CI
Batty et al. 2017					
Mesothelioma	Psychological Distress - High vs Low	HR	3.52	0.77	16.2
Bladder Cancer	Psychological Distress - High vs	HR	2.69	1.11	6.53
Leukaemia	Psychological Distress - High vs Low	HR	3.86	1.42	10.5
Liver Cancer	Psychological Distress - High vs Low	HR	4.24	0.66	8.52
Non-Hodgkin's Lymphoma	Psychological Distress - High vs Low	HR	3.14	1.36	7.24
Pancreatic Cancer	Psychological Distress - High vs Low	HR	2.76	1.47	5.19
Esophageal Cancer	Psychological Distress - High vs Low	HR	2.59	1.34	5
Multiple Myeloma	Psychological Distress - High vs Low	HR	1.95	0.25	15.5
Stomach Cancer	Psychological Distress - High vs Low	HR	2.67	1.11	6.39
Central Nervous System Cancers	Psychological Distress - High vs Low	HR	2.05	0.8	5.26
Kidney Cancer	Psychological Distress - High vs Low	HR	1.62	0.49	5.39
Other Cancers	Psychological Distress - High vs Low	HR	1.55	1.22	1.96
Pinquart et al. 2010					
Leukemia/Lymphoma	Depression	HR	1.13	1.03	1.24
Brain Cancer	Depression	HR	1.23	1.04	1.47
Other Cancers	Depression	HR	1.14	1.04	1.24
**Null**	**Factor Tested**	**Ratio Type**	**Ratio**	**Low CI**	**High CI**
Nakaya et al. 2010					
Stomach Cancer	Extraversion	HR	1	0.93	1.07
Nervous System Cancers	Extraversion	HR	1.05	0.93	1.17
Stomach Cancer	Neuroticism	HR	0.99	0.9	1.08
Nervous System Cancers	Neuroticism	HR	1.05	0.95	1.16
**Protective**	**Factor Tested**	**Ratio Type**	**Ratio**	**Low CI**	**High CI**
Batty et al. 2009					
Non-Smoking Related Cancers	IQ < 90 - Adjusted for Age and All Mediating Variables	HR	1.07	0.48	2.4
Non-Smoking Related Cancers	90 < IQ < 102 - Adjusted for Age and All Mediating Variables	HR	0.92	0.42	1.98
Non-Smoking Related Cancers	102 < IQ < 112 - Adjusted for Age and All Mediating Variables	HR	0.81	0.38	1.7
Non-Smoking Related Cancers	IQ ≥ 112 - Adjusted for Age and All Mediating Variables	HR	1	0	0
Non-Smoking Related Cancers	Per 1 SD Decrease in IQ - Adjusted for Age and All Mediating Variables	HR	1.06	0.79	1.43

Survival

Harmful	Factor Tested	Ratio Type	Ratio	Low CI	High CI
Jansen et al. 2018					
Head and Neck Cancers	Pretreatment Depressive Symptoms - Model Adjusted	HR	1.21	0.97	1.52
Head and Neck Cancers	Never Depressive Symptoms - Model Adjusted	HR	Reference	0	0
Head and Neck Cancers	Recovered from Depressive Symptoms - Model Adjusted	HR	1.06	0.62	1.83
Head and Neck Cancers	Presistent/ Recurrent/ Late Depressive Symptoms - Model Adjusted	HR	1.66	1.09	2.53

Smoking					
**Harmful**	**Factor Tested**	**Ratio Type**	**Ratio**	**Low CI**	**High CI**
Zvolensky et al. 2015					
Lifetime Cigarette Use					
Smoking	Personality Factor - Agreeableness	OR	1	0.8	1.2
Smoking	Personality Factor - Extraversion	OR	1	0.8	1.2
Smoking	Personality Factor - Neuroticism	OR	1.2	1.1	1.4
Smoking	Personality Factor - Conscientiousness	OR	0.7	0.6	0.9
Smoking	Personality Factor - Openness	OR	1.2	1	1.5
Progression to Daily Smoking					
Smoking	Personality Factor - Agreeableness	OR	1.3	0.98	1.7
Smoking	Personality Factor - Extraversion	OR	1.1	0.9	1.4
Smoking	Personality Factor - Neuroticism	OR	1.3	1.1	1.5
Smoking	Personality Factor - Conscientiousness	OR	0.7	0.5	0.9
Smoking	Personality Factor - Openness	OR	1	0.8	1.3
Persistence of Daily Smoking					
Smoking	Personality Factor - Agreeableness	OR	1	0.8	1.4
Smoking	Personality Factor - Extraversion	OR	1.1	0.9	1.5
Smoking	Personality Factor - Neuroticism	OR	1.3	1.1	1.6
Smoking	Personality Factor - Conscientiousness	OR	0.7	0.5	0.9
Smoking	Personality Factor - Openness	OR	1	0.8	1.3
